# Effectiveness of Video Podcast vs. Traditional Live Lectures in Dental Education: A Comparative Study

**DOI:** 10.12669/pjms.41.10.10817

**Published:** 2025-10

**Authors:** Rabia Anis, Salman Shafique, Faizan Alam, Hafsa Feroze

**Affiliations:** 1 Rabia Anis, (BDS, Bed, CHPE, MHPE) Senior Lecturer, Department of Health Professions, Isra Dental College, Hyderabad, Pakistan; 2 Salman Shafique, (BDS, FCPS -Oral and Maxillofacial Surgery) Professor and Dean, Department of Oral and Maxillofacial Surgery, Isra Dental College, Hyderabad, Pakistan; 3 Faizan Alam, (BDS, MDS-Oral and Maxillofacial Surgery) Senior Lecturer, Department of Oral and Maxillofacial Surgery, Isra Dental College, Hyderabad, Pakistan; 4 Hafsa Feroze, (BDS, MHPE-S) Lecturer, Department of Oral Medicine and Periodontology, Hamdard Dental Hospital, Karachi, Pakistan

**Keywords:** Dental Education, Podcast in Medical Education, Student’s Perception on Podcast Lectures, Traditional Live Lecture, Video Podcast

## Abstract

**Objective:**

To compare the effectiveness of video podcast lectures versus traditional live lectures in enhancing learning outcomes for final-year BDS students at Isra Dental College.

**Methodology:**

This quasi-experimental study was conducted over a six months and involved 49 final-year BDS students. Each student participated in both video podcast and traditional live lectures on two topics of equal difficulty, with three lectures per topic. A post-test of 20 One Best Choice Questions evaluated learning effectiveness. The same facilitator delivered both lecture formats. Data were analyzed using SPSS, with paired sample t-tests determining statistical significance (p < 0.05). Student readiness and satisfaction with video podcast lectures were assessed through pre- and post-intervention questionnaires.

**Results:**

Traditional live lectures had a significantly higher mean score (11.16) compared to video podcast lectures (9.71) with a p-value < 0.001. Before the intervention, 84% of students believed in the potential benefits of video podcasts and 79% were satisfied after the intervention. In contrast, 95% were satisfied with traditional lectures. Although 90% of students were familiar with podcasting, mostly using mobile phones (65%), 84% still preferred podcasts over traditional lectures. Post-intervention feedback indicated high satisfaction with the tools, sound quality, synchronization and engagement of video podcasts.

**Conclusions:**

Traditional live lectures were more effective in improving learning outcomes. However, video podcasts were valued for their convenience and the ability to review content. Integrating both formats could better accommodate diverse learning preferences and enhance the educational experience.

## INTRODUCTION

Information technology has significantly impacted higher education, including medical and dental education.^1^ Over the past decade, educators have increasingly adopted digital tools to enhance teaching and learning. Among these advancements, video podcasts have gained popularity as a versatile and accessible learning resource.^1^ Video podcasts typically combine audio recordings with video images, such as PowerPoint slideshows, offering several potential benefits for students.^2^

One key advantage of video podcasts is the flexibility they provide, allowing students to learn at their own pace by pausing, rewinding and reviewing material as needed. This is particularly valuable in complex subjects like dentistry, where mastery of specific concepts often requires repeated study.^3^ The convenience of accessing lectures from any location and at any time is especially beneficial for students with busy schedules or other commitments.^4^

Additionally, video podcasts can serve as supplementary resources to traditional teaching methods, reinforcing students’ learning. From a pedagogic perspective, video podcasts align with Mayer’s multimedia learning theory, which posits that effective learning occurs through dual channels visual and auditory. When both channels are stimulated, learning is enhanced. The ability to pause and repeat podcast content supports retention by addressing the limited capacity of working memory. Moreover, active processing, which involves organizing and integrating new information with existing knowledge, further enhances learning.^3^

Having these advantages, video podcast lectures also have drawbacks. Since it may result in more passive learning, the reduced interaction between the teacher and students is a major concern. Traditional live lectures allow for immediate questions and feedback, which improves understanding and engagement.^5^ The absence of nonverbal cues in video podcasts may also hinder lecturers’ ability to gauge students’ comprehension.^6^ The potential of video podcasts to provide an engaging learning environment may be diminished by these factors.^7^

The use of video podcasts in medical, dental and nursing education has grown steadily in recent years.^7^ However, evidence suggests that traditional in-person lectures may still hold advantages in certain contexts. For instance, AlKahtani et al. (2025), in a meta-analysis comparing live and video-based demonstrations, concluded that while digital formats offer flexibility, live lectures result in greater learner engagement and improved performance.^8^ Similarly, it was observed that students found podcasts helpful for review but preferred face-to-face instruction for learning new material due to its interactive nature.^9^

Additionally, Alshaikhi S et al. confirmed through a scoping review that podcasts play a valuable role in medical education, particularly when integrated thoughtfully into blended learning models.^10^ As educational institutions increasingly adopt blended learning approaches that combine traditional and digital teaching methods, it is essential to evaluate how effectively video podcasts complement live lectures. The findings of this study aimed to assess whether video podcasts can serve as a valuable supplement, ultimately enriching the educational experience of dental students. By providing comparative evidence on podcast-based and traditional learning strategies in a low-resource setting, this study contributes to the growing body of literature on blended learning in dental education. It further highlights students’ preferences and the practical feasibility of integrating podcasts as supplementary learning tools.

## METHODOLOGY

This quasi-experimental study was conducted over a six months period at Isra Dental College, Hyderabad, involving all final-year BDS students (N=49). The primary aim was to compare the effectiveness of video podcast lectures versus traditional live lectures on two topics of equal difficulty, as determined by three content experts. Each topic was covered in three lectures, delivered by the same facilitator for consistency. To ensure the validity of results, the same content experts also prepared and reviewed the examination papers and assessment materials.

### Ethical approval:

The study received ethical approval from the Ethics Committee of Isra University (Ref. No.: IU/IDC/R.A/2023/448; Date: August 30, 2023) and informed consent was obtained from all participants prior to the commencement of the study.

The study utilized convenience sampling, involving 24 male and 25 female students, with a mean age of 23 ± 1.5 years. The sample size was determined based on the total number of final-year students available at the time of the study, ensuring that all eligible students were included. While a formal sample size calculation was not conducted due to the use of convenience sampling, the entire cohort of students was selected to maximize the study’s power and representativeness.

The video podcast lectures combined audio recordings with PowerPoint slides, created using Microsoft PowerPoint and recorded with Camtasia software. Traditional live lectures were delivered in a classroom setting by the same facilitator. To evaluate learning effectiveness, post-tests consisting of 20 One Best Choice Questions per topic were administered immediately after each set of lectures. These tests assessed students’ understanding and retention of the material. Data were collected through computerized scanning of the answer sheets and analyzed using SPSS version 25. Paired sample t-tests were used to compare the mean scores between the video podcast and traditional live lecture groups, with a p-value <0.05 considered statistically significant.

Student readiness for video podcast lectures was assessed through a pre-intervention questionnaire, which included Likert-scale questions about previous podcast experience and expectations. The post-intervention questionnaire evaluated satisfaction, convenience and the ability to review material, using a similar Likert scale to rate the overall experience, appropriateness of the podcast tool and engagement.

Perceptions of traditional lectures were not assessed separately, as students were already familiar with this format and regular feedback is collected through the existing system. This approach focused the study on the novel aspect of video podcast lectures, avoiding redundancy.

## RESULTS

The study assessed the effectiveness of learning through post-test scores consisting of 20 One Best Choice Questions for each topic. The traditional live lecture group demonstrated a significantly higher mean score compared to the video podcast lecture group. The mean score for the traditional live lecture group was 11.16, whereas the mean score for the video podcast lecture group was 9.71. The difference in mean scores was statistically significant (p < 0.001), indicating that traditional live lectures had a higher educational impact than video podcast lectures (Table-I). The cut-off p-value for determining statistical significance was set at 0.05.

**Table-I:** Comparison of Learning Outcomes.

Lecture Type	Mean Score	Standard Deviation	Standard Error of Mean
Video Podcast	9.71	3.657	0.522
Traditional Live	11.16	3.299	0.471

The pre-intervention survey showed that 84% of students believed that video podcast lectures could be beneficial for their learning. Post-intervention feedback revealed that 79% of students were satisfied with the video podcast lectures. In comparison, 95% of students expressed satisfaction with traditional live lectures (Table-II).

**Table-II:** Student Satisfaction and Belief in Benefit.

Lecture Type	Student Satisfaction (%)	Student Belief in Benefit (%)
Video Podcast	79	84
Traditional Live	95	92

The pre-intervention questionnaire results indicate that a majority of students (90%) are familiar with podcasting and predominantly use mobile phones (65%) to access podcasts. While 40% of students rarely download podcasts, 40% do so regularly or often. Despite limited prior exposure to podcast lectures, with 50% having attended none, 84% believe that podcasts could be beneficial. Around 80% of students prefer podcast lectures over traditional live lectures, although their knowledge about podcasting technology varies. Awareness of peers’ experiences with podcast lectures is relatively low at 20%. These findings suggest a positive attitude towards podcasting as a learning tool, highlighting the need for further integration and support in educational settings (Table-III that illustrates student familiarity and access preferences).

**APPENDIX: Table-III:** Pre-Intervention Questionnaire Results.

Question	Response Options	Percentage (%)
Familiarity with podcasting	Yes	90
	No	10
Devices used for podcasts	Mobile Phone	65
	Personal Computer	15
	All of the above	10
	Video Player	10
Frequency of downloading podcasts	Regular	20
	Often	20
	Rare	40
	Never	20
Number of podcast lectures attended	None	50
	1-5	20
	5-10	10
	More than 10	20
Belief in benefits of podcasting	Yes	84
	No	10
	Maybe	6
Preference for podcast lectures over traditional	Yes	80
	No	15
	Maybe	5
Knowledge about podcasting technology	Knowledgeable	25
	A little knowledgeable	50
	Neutral	15
	Not at all knowledgeable	10
Awareness of peers’ experiences with podcasts	Yes	20
	No	80

The post-intervention questionnaire results indicate that the majority of students had a positive experience with the video podcast lectures, with high satisfaction ratings for the selection of tools, quality of sound and synchronization of the facilitator’s voice with the content. Engagement levels were also rated favorably. While a significant number of students believed that video podcasts have a positive learning impact, opinions were mixed on whether traditional lectures should be replaced entirely by video podcasts. These results highlight the potential of video podcasts as a supplementary educational tool, suggesting that a blended learning approach might be the most effective way to cater to diverse student preferences and maximize learning outcomes (Table-IV that highlights post-intervention satisfaction trends).

**Table-IV:** Post-Intervention Questionnaire Results.

Question	Very Satisfied	Satisfied	Neutral	Dissatisfied	Very Dissatisfied
How do you rate your experience of podcast lecture?	30%	35%	20%	10%	5%
Was the selection of tool for Video podcast appropriate?	40%	30%	25%	3%	2%
Was the quality of Video Podcast sound reasonable?	35%	30%	20%	10%	5%
Was the voice of a facilitator in Video podcast lecture synchronized with the content?	40%	35%	20%	5%	-
How would you rate the level of engagement in video podcast lecture?	30%	35%	25%	5%	5%
Do you believe that video podcast has a learning impact?	40%	30%	20%	5%	5%
Do you think in future, traditional class format lecture should be replaced with video podcast lecture?	20%	15%	30%	20%	15%

## DISCUSSION

This study found that traditional live lectures resulted in significantly higher post-test scores compared to video podcast lectures among final-year dental students. While students appreciated the flexibility and convenience of podcasts, they expressed a preference for the interactive nature of live teaching. These findings are consistent with national and international studies. For example, McCarthy et al. (2023) reported that students found podcasts helpful for reviewing missed content but preferred live sessions for initial learning due to the opportunity for real-time interaction.^7^ Similarly, AlKahtani et al. (2025) conducted a meta-analysis and concluded that live instructional formats were more effective for engagement and performance than video-based formats.^8^ Similar to our findings, Gardiakos et al. (2025) emphasized that podcasts, when integrated as supplementary resources, can enhance learning flexibility and engagement, particularly when aligned with clear learning outcomes and evaluated for discipline-specific effectiveness.^9^

Regionally, Alshaikhi et al. & Madini et al. (2016) studied medical students in Saudi Arabia and observed that video podcasts were perceived as beneficial for revision but lacked the engagement factor of live lectures.^10^ Internationally, Allen et al. (2011) highlighted in a scoping review that the integration of podcasts is most effective when embedded within broader blended learning strategies.^11^ Globally, multiple studies support a blended learning approach. Guo et al. (2014) noted that while students appreciated the ability to review lectures at their own pace, the absence of real-time interaction in podcasts could hinder engagement and deeper comprehension.^12^ Guo et al. (2014) further highlighted that students engage more effectively when video lectures are combined with interactive elements, emphasizing the need for a hybrid approach.^12^

One of the primary reasons why live lectures do better than video podcasts could be lower motivation and discipline in self-paced learning environments may affect students’ ability to engage in audio lectures.^13^ Additionally, other factors could be the lack of immediate interaction and feedback, the dynamic and interactive character of live lectures allowing for real-time inquiries and explanations, which facilitates a deeper understanding and retention of complex idea.^14^,^15^ Another factor that might have prevented students from making the most of the format was their inexperience with effective podcast-based learning strategies. The presenting style and technical clarity of video podcasts can have a significant impact on their effectiveness.^16^

These results imply that a blended learning strategy that combines both traditional live lectures and video webcast lectures could maximize student learning experiences in terms of clinical importance. Video podcasts offer flexibility and the opportunity to repeat material for improved retention, while traditional lectures offer interactive learning and a basic understanding. In the end, this hybrid approach improves dentistry students’ entire educational experience by being in line with contemporary teaching strategies that accommodate a variety of learning preferences.

### Limitations:

This study was conducted at a single institution with a modest sample size, which may affect generalizability. While formal randomization was not applied, the within-subjects design ensured consistency. MCQs assessed knowledge but may not reflect deeper learning. Despite these constraints, the study provides meaningful insights into instructional strategies in dental education and supports future exploration.

This study adds to the existing body of literature by providing evidence from a low-resource setting, directly comparing video podcast lectures with traditional live teaching among final-year dental students. It highlights that, while students value the flexibility of digital tools, traditional lectures still play a vital role in ensuring effective knowledge acquisition. The findings underscore the importance of active, interactive instruction in dental education and suggest that digital learning tools should complement not replace traditional methods.

### Strength:

The clinical relevance lies in its implications for curriculum design, particularly in enhancing lecture-based teaching to improve knowledge retention and student satisfaction. A major strength of this study is its within-subject design, which minimized instructor variability by using the same facilitator for both modes of delivery. Additionally, the study employed a structured pre- and post-intervention assessment with validated questionnaires.

## CONCLUSIONS

This study has shown that traditional live lectures are more effective than video podcast lectures in enhancing the learning outcomes of final year dental students at Isra Dental College. However, video podcasts were highly valued for their convenience and ability to facilitate content review. These findings suggest that a blended learning approach, incorporating both traditional and video podcast lectures, may be the most effective strategy for maximizing educational outcomes and student satisfaction in dental education.

### Recommendations:

Future studies should explore the impact of video podcasting on long-term knowledge retention, clinical skill performance and how digital learning tools can be adapted for greater interactivity and engagement. Qualitative insights may further enrich understanding of student learning preferences in diverse educational environments.
